# Loquat (*Eriobotrya japonica*) is a New Natural Host of Apple Stem Pitting Virus

**DOI:** 10.3390/plants9111560

**Published:** 2020-11-13

**Authors:** Félix Morán, Celia Canales, Antonio Olmos, Ana Belén Ruiz-García

**Affiliations:** Centro de Protección Vegetal y Biotecnología, Instituto Valenciano de Investigaciones Agrarias (IVIA), Ctra. Moncada-Náquera km 4.5, Moncada, 46113 Valencia, Spain; moran_fel@gva.es (F.M.); canales_cel@externos.gva.es (C.C.); aolmos@ivia.es (A.O.)

**Keywords:** ASPV, HTS, loquat

## Abstract

Loquat (*Eriobotrya japonica*) is a minor but important woody crop cultivated in Asia and Europe. High-throughput sequencing (HTS) analysis of an asymptomatic loquat plant using RNAseq Illumina technology has allowed the detection for the first time of apple stem pitting virus (ASPV), the type species of the genus *Foveavirus* in the family *Betaflexiviridae*, infecting this crop. A nearly complete genome of 9303 nts (ASPV-SL61) reconstructed bioinformatically shows the typical genomic structure of this viral species and a highest nucleotide identity (85.9%) with the Chinese ASPV isolate YLX from pear. A close phylogenetic relationship between ASPV-SL61 and ASPV-YLX has been confirmed by the sequence analysis of full-length ASPV genomic sequences available in the databases. In fact, a phylogenetic study based on a partial CP N-terminal sequence previously proposed to be involved in host adaptation has shown that ASPV-SL61 loquat isolate is more closely related to ASPV pear isolates. The presence of ASPV in loquat has been further confirmed by RT-PCR and Sanger sequencing and DAS-ELISA. An incidence of 15% was determined in one of the loquat Spanish growing areas. The sequence analysis of the partial CP sequences amplified by RT-PCR has shown a high level of variability between loquat isolates. To our knowledge, this is the first record of loquat as a natural host of ASPV.

## 1. Introduction

Apple stem pitting virus (ASPV) is the type species of the genus *Foveavirus*, family *Betaflexiviridae*, subfamily *Quinvirinae* [1]. ASPV has been reported to be widely distributed and to infect several hosts including apple, pear, hawthorn, quince, cherry, and nanking cherry [2,3,4,5]. Although ASPV has been related to a broad range of symptoms such as green crinkle, star crack, epinasty, decline and stem pitting in apple, vein yellowing, leaf red mottling and necrotic spotting in pear, and fruit deformations in quince, its infection has frequently been reported to remain asymptomatic [6,7,8]. Several studies have shown a high degree of intraspecies diversity for ASPV [6,8,9]. This genetic diversity has been proposed to be host-related and to play a role in new host adaptation [8,9,10].

Loquat (*Eriobotrya* *japonica*), which belongs to the family *Rosaceae*, is a tree native to China cultivated in Asia and Europe and appreciated for its early sweet fruit and medicinal properties [11]. Spain is the main loquat exporter with an annual production of 40,000 tones representing a valuable local economic income in some regions of the country [12]. To date, only three viral pathogens have been reported to infect loquat, apple chlorotic leafspot virus (ACLSV), apple stem grooving virus (ASGV), and loquat virus A (LoVA) [11,13]. Although none of these viruses have been clearly associated with any particular disease or reduced fruit production, emergence of new viral pathogens and/or synergism between different viral species might negatively affect this crop.

With the aim of preventing the eventual emergence of viral diseases in loquat, several trees from a loquat growing area in the eastern region of Spain were analyzed by high-throughput sequencing (HTS), RT-PCR, and DAS-ELISA. This study has allowed the identification for the first time of apple stem pitting virus (ASPV) infecting loquat, providing new insights into the host range of this viral pathogen and contributing to a better understanding of the sanitary status of this crop.

## 2. Results

**Figure 1:**
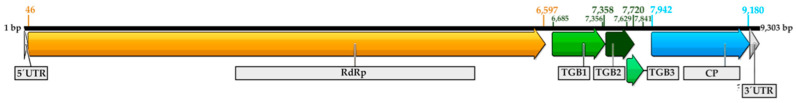
Genome structure of ASPV-SL61. Sequence length and genomic positions of the five ORFs (RdRp, TGB1, TGB2, TGB3, and CP), as well as the 5′ and 3′ UTR are indicated.

HTS analysis on total RNA extracted from an asymptomatic loquat plant (SL61, cv. Algerie) from a private garden in Segorbe, Spain, yielded 33,340,022 reads (144.2 nts average size) after trimming and quality control. A host genome subtraction step was performed, and the 3,251,340 remaining reads subjected to de novo assembly. BLASTN/X analysis of the 18,908 contigs obtained revealed the presence of 1 long contig related to ASPV. The contig of 9303 nt in length was covered by 6622 reads with an average coverage of 106.5x and comprised the full-length coding sequence as well as 45 nt 5’UTR and 123 nt 3′UTR regions (ASPV-SL61, accession number MW045213). The isolate ASPV-SL61 showed 85.9% nucleotide identity with the Chinese isolate YLX from pear (KY798310) and shared the typical genome structure of other foveaviruses (Figure 1) encoding five ORFs, the RNA dependent RNA polymerase, RdRp (2183 aa), the triple gene block proteins, TGB1 (224 aa), TGB2 (121 aa), and TGB3 (71 aa) and the coat protein, CP (413 aa). The amino acid identity percentage observed between ASPV-SL61 and ASPV-YLX at the different ORFs were 92.9% (RdRp), 98.2% (TGB1), 93.3% (TGB2), 97.2% (TGB3), and 87.7% (CP). The BLASTN/X analysis performed did not show the presence in SL61 of any other viral-related sequences.

**Figure 2:**
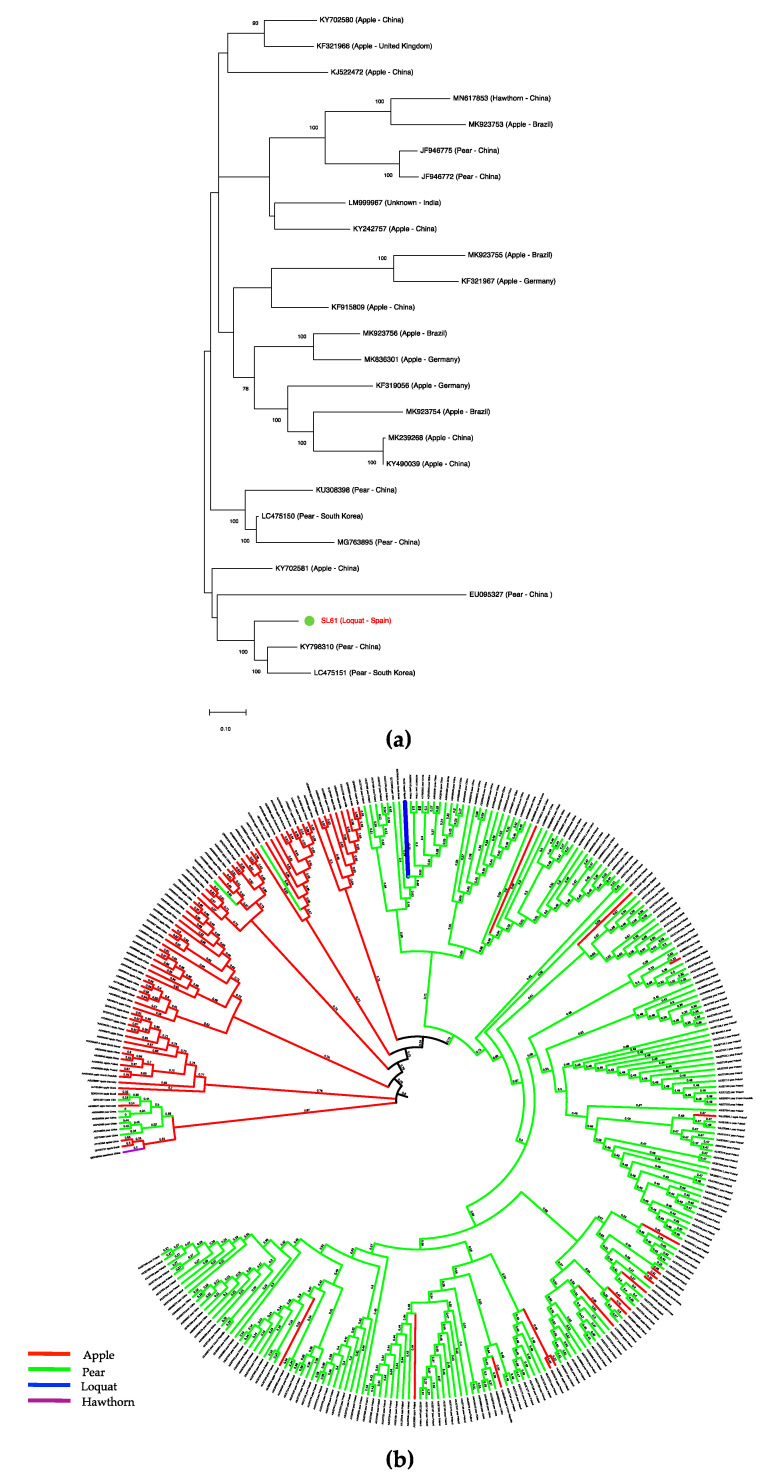
(**a**) Maximum likelihood phylogenetic tree using the best substitution model of full-length ASPV genomic sequences. Accession numbers, host, and origin are indicated. The scale bar shows the genetic distance. Bootstrap percentage are indicated on the branches. (**b**) Phylogenetic inference for molecular evolution of 250 aa N-terminal CP region by PhyML3.0 using the best substitution model. Sequences are identified by their accession numbers. Substitutions per site are indicated on the branches. Hosts are indicated in colors.

A phylogenetic analysis based on full-length sequences available in the databases showed that ASPV-SL61 isolate is phylogenetically related to pear isolates of China (isolate YLX) and South Korea (isolate WH, LC475151) with high bootstrap support (Figure 2), in agreement with the nucleotide similarity observed between SL61 and YLX by BLASTN/X analysis. 

A subsequent sequence analysis using a 250 aa CP partial sequence corresponding to the N-terminal region of the coat protein from 303 different ASPV isolates available in the databases, most of them from apple and pear (Table S1), as well as ASPV-SL61 was performed (Figure 2). This part of the CP has been described to be the most variable region of the protein and it is supposed to be involved in host adaptation [8,10]. The phylogenetic tree constructed based on this N-terminal CP sequence alignment showed that ASPV isolates are predominantly grouped by host as previously described [8,9]. ASPV CP N-terminal region grouped in two clusters, one corresponding to the majority of isolates infecting apple and a second one related to most of the isolates infecting pear, although some apple isolates clustered with pear isolates and vice versa. Isolate SL61 from loquat grouped into the main pear cluster. Several deletions on the N-terminal region of the CP respect to the isolate PA66 (NC_003462) have also been involved in ASPV host adaptation [10]. Three deletions (3, 5, and 1 nts) and one insertion (3 nts) have been found in this genomic region of ASPV-SL61. 

A recombination analysis using ASPV full-length sequences available in the databases as well as ASPV-SL61 was performed. A potential recombination event was found in ASPV SL-61 between genomic positions 6625 and 7878 (99% CI) by seven algorithms (*p*-values 4.36 × 10^−262^, 1.00 × 10^−173^, 1.28 × 10^−263^, 7.25 × 10^−50^, 6.18 × 10^−57^, 6.02 × 10^−62^, and 4.15 × 10^−241^, for RDP, GENECONV, BootScan, MaxChi, Chimaera, SiScan, and 3Seq, respectively). All software suggested as minor parent EU095327 and unknown major parent. 

The presence of ASPV in sample SL61 was confirmed by RT-PCR. A region of 385 nts containing the C-terminal region of the CP and part of the 3′UTR was successfully amplified using previously reported ASPV specific primers [14] despite discrete mismatching in the primer sequence with respect to the HTS determined sequence. Sanger sequencing of the amplicon obtained confirmed the ASPV-SL61 sequence recovered by HTS in this genomic region. To further confirm loquat as a new host of ASPV, a random survey was conducted in the same geographical area. In order to improve ASPV detection in loquat orchards, new primers amplifying the same genomic region than those previously described [14] were designed based on a perfect alignment on the loquat isolate ASPV-SL61. Among a total of 80 plants analyzed by RT-PCR, 10 plants tested positive for ASPV. It is important to note that no virus-like symptoms were observed in any of these analyzed plants.

**Figure 3:**
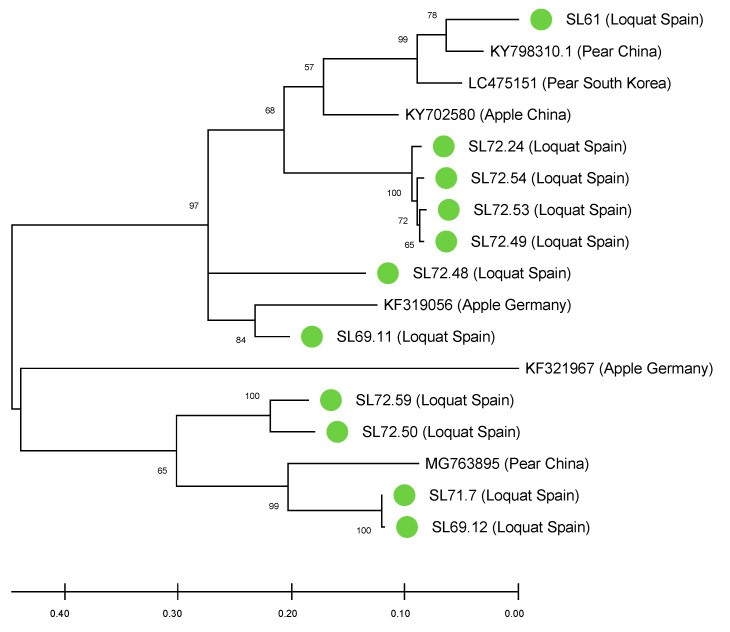
Maximum likelihood phylogenetic tree using the best substitution model of loquat CP partial genomic sequences. Accession numbers (for database available sequences) or code sample (isolate names for sequences obtained in this study), host, and origin are indicated. The scale bar shows the genetic distance. Bootstrap percentage are indicated on the branches.

To evaluate the sequence variability among the Spanish loquat isolates detected, a phylogenetic analysis was conducted based on this CP partial sequence. This analysis revealed a high level of genetic diversity between loquat isolates, which were grouped in two different clusters related to ASPV sequences from both pear and apple (Figure 3). Future studies are needed to certainly assess the variability observed, by generating more exhaustive molecular data. 

The presence of ASPV in loquat was also assessed by ELISA. Among the 80 loquat plants surveyed, 10 plants tested positive for ASPV (Table 1). The comparison between the results obtained by the two detection techniques (RT-PCR and DAS-ELISA) showed that 3 of the plants that tested positive by RT-PCR were not detected by ELISA. On the other hand, the ELISA method allowed the detection of 2 ASPV positive samples that had tested negative by RT-PCR. A total incidence of 15% for ASPV in this loquat growing area was calculated by the combination of both techniques.

## 3. Discussion

HTS has become a powerful tool in plant pathology research, allowing the detection and characterization of both known and unknown viruses infecting a plant, and thus giving a global picture of the plant virome [15,16]. To date, very few studies on loquat virome have been reported. In fact, only three viruses are known to infect this crop, ACLSV, ASGV, and LoVA. Furthermore, there is a complete lack of knowledge on the loquat virome in Spanish orchards, where loquat is a minor but important crop. In this study, with the aim of increasing our knowledge on loquat virome as well as evaluating the potential threat of emerging viruses infecting loquat in Spain, HTS has been applied. The HTS analysis performed in this work on an asymptomatic loquat plant from a loquat Spanish growing area has revealed this crop as a natural host susceptible to be infected by another virus, ASPV. The isolate ASPV-SL61 shares the highest nucleotide and aminoacid identity with ASPV-YLX, a Chinese isolate found in pear. In fact, a sequence analysis performed on all the ASPV full-length isolates available in the databases has shown a close phylogenetic relationship between these two isolates. No other viral-related sequences were detected by HTS in SL61 sample. To our knowledge, this is the first record of ASPV infecting loquat, opening new insights into the host range of this viral species.

A sequence analysis was performed using 303 APSV isolates from different hosts and origins on the N-terminal region of the CP, previously described to be related to host infecting ability of the virus [8,9]. Two big clusters were observed in the phylogenetic tree constructed, grouping most pear or apple isolated variants respectively, according to the phylogenetic grouping by host previously reported [8,9]. Interestingly, ASPV-SL61 loquat isolate grouped into the pear cluster, in agreement with the phylogenetic analysis conducted on full-length genomic sequences. Deletions in the N-terminal region of the CP gene have been proposed to play a role in host selection [10]. Three deletions and one insertion were found in the ASPV-SL61 N-terminal CP region respect to the AP66 isolate, which might be involved in loquat adaptation. Further molecular characterization of ASPV isolates from quince, hawthorn, cherry, and loquat, for which limited sequence data are available, would contribute to a better understanding of the genomic features determining the ability of ASPV to infect different hosts.

Recombination has been shown to be an important driving force in ASPV evolution [11,17,18]. The results of the recombination analysis performed in this study has shown additional support for a relationship between loquat ASPV and pear isolates. In fact, strong evidence for a recombination event in the TGB region of ASPV-SL61 has been found, probably involving as minor parent a pear isolate from China (isolate PR1, EU095327). 

Loquat has been confirmed as a new natural host of ASPV by RT-PCR targeting a partial region of the CP and by DAS-ELISA analysis of 80 loquat samples collected in a random survey conducted in a Spanish loquat growing area, where a 15% ASPV incidence has been found. Interestingly, among the 12 ASPV positive samples detected, 5 samples showed a result discrepancy between the two detection methods. In particular, 3 samples that tested positive by RT-PCR were not detected by ELISA, probably due to a lower sensitivity of the serological detection method. The opposite situation was also observed, 2 samples that tested positive by ELISA were not detected by RT-PCR. In this case, the disagreement between methods could be due to the genetic diversity observed between the Spanish isolates based on the phylogenetic analysis conducted on the CP region. It is important to note that none of the positive plants detected showed any virus-like symptomatology, which seems to indicate an asymptomatic infection of ASPV in loquat. In this sense, symptomless ASPV infections have frequently been reported in the literature [6,7,8]. 

The sequence analysis on the CP of the ASPV infected plants has revealed a high degree of variability between isolates that are phylogenetically grouped in at least two different clusters. This genetic diversity raises two important issues to be investigated: (i) the origin of the intraspecies variability shown by ASPV loquat isolates, taken into account that no ASPV vector has been described to date and therefore that viral spread seems to take place through mechanical transmission; and (ii) the implications of this variability for the reliability of ASPV diagnosis in this crop. Future studies on different loquat growing areas and cultivars providing new molecular data will help to elucidate these questions.

Despite the absence of symptomatology in the loquat plants monitored in this study, the existence of aggressive ASPV variants, the presence of other viral species, and/or putative synergism between pathogens causing a significant impact on loquat viability and production cannot be excluded. Our results point to the need of a more in-depth study on loquat virome with the aim of preventing putative emerging viral diseases that might have a negative impact on this crop.

## 4. Materials and Methods 

### 4.1. Plant Material

A total of 81 loquat leaf samples (cv. Algerie) collected from small familiar orchards in Segorbe, eastern region of Spain, were used in this study. Sample SL61 was collected from a private garden in the same geographical location.

### 4.2. Sample Preparation and RNA Purification

Leaf tissue was placed in plastic bags (Bioreba, Reinach, Switzerland) and extraction buffer (PBS containing 0.2% diethyldithiocarbamate and 2% PVP-10) was added up to a 1:5 ratio (*w*:*v*). Homex 6 homogenizer (Bioreba, Reinach, Switzerland) was used for grinding. For DAS-ELISA test 200 µL of extract was used. Total RNA was purified using the Plant/Fungi Total RNA Purification Kit (Norgen Biotek Corporation, Thorold, ON, Canada) following manufacturer instructions. RNA was quantified with a DeNovix DS-11 spectrophotometer (DeNovix Inc., Wilmington, DE, USA) to determine RNA concentrations and stored at −80 °C until subsequent analysis. 

For HTS analysis, complementary DNA was synthesized and the sequencing library prepared using the TruSeq Stranded Total RNA LT Sample Prep Kit (Plant) and the library protocol TruSeq Stranded Total RNA Sample Prep Guide, Part #15031048 Rev. E. RNA quality control, library construction and sequencing in a NextSeq 500 platform (paired 2x150nt) were performed at Macrogen Inc. (Seoul, Republic of Korea).

### 4.3. Bioinformatic Analysis of HTS Data

Raw reads were subjected to trimming of adapters and quality control using CLC Genomics Workbench v.20.0.4 (Qiagen Bioinformatics, Hilden, Germany). *Eriobotrya* *japonica* genome GWHAAZU00000000 [19] and *Eriobotrya* *japonica* mitochondrion (NC_045228) and chloroplast (NC_034639) complete genome were used for genome subtraction. De novo assembly was performed using CLC Genomics Workbench v.20.0.4 and de novo contigs larger than 200 nt were annotated by BLAST analysis (BLASTN/X) with a cut-off e-value of 10^−4^ against local and online virus, viroids, and nt/nr databases.

### 4.4. ASPV Detection by RT-PCR

ASPV was detected by RT-PCR using previously described primers qASP-F (5′-TGC CTT TTA CGC AAA GCA TGT-3′, sense) and ASPV-R (5′-TTG GGA TCA ACT TTA CTA AAA AGC ATA A-3′, antisense) [14] amplifying 385 bp between the C-terminal region of the CP and the 3′UTR and two newly designed primers based on the HTS recovered sequence qASP-FS (5′-CGC TTT CTA CGC GAA GCA TGT-3′, sense) and ASPV-RS (5′-TTG GGA TCA ACT TTA TTA AAA GCA TAA-3′, antisense) amplifying the same genomic region. RT-PCR analysis was performed using AgPath-ID One-Step RT-PCR Kit (Ambion Inc., Austin, TX, USA) following the manufacturer instructions. The reaction mixture contained 0.4 μM of each of the primers and 3 μL of sample containing 50 ng of total RNA. RT-PCR protocol consisted of one step of 45 °C for 30 min and one step of 95 °C for 10 min, followed by 40 cycles of amplification (95 °C for 30 s, 50 °C for 30 s, and 60 °C for 1 min). Amplicons were purified using mi-PCR Purification Kit (Metabion International AG, Martinsried, Germany), and Sanger sequenced in both directions.

### 4.5. ASPV Detection by DAS-ELISA

ASPV was detected by DAS-ELISA using the ASPV Complete Kit (Bioreba, Reinach, Switzerland) following the manufacturer instructions. Two replicates of each sample were analyzed. Color reaction was monitored measuring the absorbance at 415 nm after two hours, using an iMark Microplate Reader (Bio-Rad Laboratories, Inc., Hercules, CA, USA). Results were considered positive when the absorbance value was equal to or greater than twice the absorbance of a negative control (plant extract from ASPV-free loquat plant.

### 4.6. Sequence Alignment and Phylogenetic Analysis

Nearly complete ASPV-SL61 nucleotide genomic sequence and 25 full-length ASPV genomes from different hosts and origins (KY798310, JF946772, JF946775, KF319056, KF321966, KF321967, KF915809, KJ522472, KU308398, KY242757, KY490039, KY702580, KY702581, LC475150, LC475151, LM999967, MG763895, MK239268, MK836301, MK923753, MK923754, MK923955, MK923756, EU095327, and MN617853) were aligned using the ClustalW implemented in MEGA X [20]. The 11 partial CP sequences from loquat isolates generated in this study were also aligned as described above. The phylogenetic trees were constructed with the maximum likelihood algorithm implemented in MEGA X applying the lowest BIC (Bayesian information criterion), the general time reversible model [21] with rates among sites gamma distributed and with invariant sites (G+I), in the case of the full-length sequences, and the Tamura 3-parameter model [22] with rates among sites gamma distributed and with invariant sites (G+I), in the case of the CP partial sequences, and with 500 bootstraps.

For the phylogenetic analysis based on the N-terminal aminoacidic region (250 aa) of the CP from 303 isolates (Table S1), sequences were aligned using ClustalW implemented in Geneious Prime 2020 (Biomatters Ltd., Auckland, New Zealand). The phylogenetic tree was constructed by PhyML3.0 [23] implemented in Geneious using the best substitution model, the Jones–Taylor–Thornton model [24] with rates among sites gamma distributed and with invariant sites (G+I), and with 100 bootstraps. 

### 4.7. ASPV Recombination Analysis

ASPV-SL61 and 25 full-length ASPV genomes available in the databases (KY798310, JF946772, JF946775, KF319056, KF321966, KF321967, KF915809, KJ522472, KU308398, KY242757, KY490039, KY702580, KY702581, LC475150, LC475151, LM999967, MG763895, MK239268, MK836301, MK923753, MK923754, MK923955, MK923756, MN617853, and EU095327) were analyzed for potential recombination events using RDP v.4.1 with default settings. A cut-off p-value lower than 0.001 for more than five algorithms was applied for considering a recombination event.

## Figures and Tables

**Table 1:** Comparison of the ASPV diagnostic results obtained by RT-PCR and DAS-ELISA for the 13 loquat infected plants detected in this study. Sample code as well as positive (+) or negative (−) detection by each method are indicated.

Sample Code	RT-PCR	DAS-ELISA
SL 61	+	+
SL 69.11	+	+
SL 69.12	+	−
SL 71.7	+	+
SL 72.24	+	−
SL 72.48	+	+
SL 72.49	+	+
SL 72.50	+	+
SL 72.53	+	−
SL 72.54	+	+
SL 72.59	+	+
SL 72.1	−	+
SL 72.22	−	+
**Total positives**	**11**	**10**

## Supplementary Materials

The following are available online at https://www.mdpi.com/2223-7747/9/11/1560/s1, Table S1: Accession numbers, host and origin for sequences used in the phylogenetic analysis performed on the CP N-terminal region.

## References

[1] Martelli G.P., Jelkmann W. (1998). Foveavirus, a new plant virus genus. Arch. Virol..

[2] Mathioudakis M.M., Maliogka V.I., Dovas C.I., Vasilakakis M., Katis N.I. (2006). First record of the apple stem pitting virus (ASPV) in quince in Greece. J. Plant Pathol..

[3] Dhir S., Tomar M., Thakur P.D., Ram R., Hallan V., Zaidi A.A. (2010). Molecular evidence for apple stem pitting virus infection in India. Plant Pathol..

[4] Yang H.Y., Liu Z.J., Luo S., Li L.L. (2017). First report of apple stem pitting virus infecting nanking cherry in China. Plant Dis..

[5] Xing F., Hou W., Massart S., Gao D., Li W., Cao M., Zhang Z., Wang H., Li S. (2020). RNA-seq reveals hawthorn tree as a new natural host for apple necrotic mosaic virus, possibly associated with hawthorn mosaic disease. Plant Dis..

[6] Mathioudakis M.M., Maliogka V.I., Katsiani A.T., Katis N.I. (2010). Incidence and molecular variability of apple stem pitting and apple chlorotic leaf spot viruses in apple and pear orchards in Greece. J. Plant Pathol..

[7] Wu Z.B., Ku H.M., Su C.C., Chen I.Z., Jan F.J. (2010). Molecular and biological characterization of an isolate of apple stem pitting virus causing pear vein yellows disease in Taiwan. J. Plant Pathol..

[8] Ma X., Hong N., Moffett P., Zhou Y., Wang G. (2019). Functional analysis of apple stem pitting virus coat protein variants. Virol. J..

[9] Ma X., Hong N., Moffett P., Wang G. (2016). Genetic diversity and evolution of apple stem pitting virus isolates from pear in China. Can. J. Plant Pathol..

[10] Komorowska B., Hasiów-Jaroszewska B., Elena S.F. (2019). Evolving by deleting: Patterns of molecular evolution of apple stem pitting virus isolates from Poland. J. Gen. Virol..

[11] Liu Q., Xuan Z., Wu J., Qiu Y., Li M., Zhang S., Wu D., Li R., Cao M. (2019). Loquat is a new natural host of apple stem grooving virus and apple chlorotic leaf spot virus in China. Plant Dis..

[12] Caballero P., Fernández M.A., Llácer G., Badenes M.L. (2003). Loquat, production and market. Options Méditerranéennes: Série A. Séminaires Méditerranéens n. 58, Proceedings of the First International Symposium on Loquat, Zaragoza, Spain, 11–13 April 2002.

[13] Liu Q., Yang L., Xuan Z., Wu J., Qiu Y., Zhang S., Wu D., Zhou C., Cao M. (2020). Complete nucleotide sequence of loquat virus A, a member of the family Betaflexiviridae with a novel genome organization. Arch. Virol..

[14] Malandraki I., Beris D., Isaioglou I., Olmos A., Varveri C., Vassilakos N. (2017). Simultaneous detection of three pome fruit tree viruses by one-step multiplex quantitative RT-PCR. PLoS ONE.

[15] Maliogka V.I., Minafra A., Saldarelli P., Ruiz-García A.B., Glasa M., Katis N., Olmos A. (2018). Recent advances on detection and characterization of fruit tree viruses using high-throughput sequencing technologies. Viruses.

[16] Villamor D.E.V., Ho T., Al Rwahnih M., Martin R.R., Tzanetakis I.E. (2019). High throughput sequencing for plant virus detection and discovery. Phytopathology.

[17] Dhir S., Ram R., Hallam V., Zaidi A.A. (2011). Molecular characterization of an Indian variant of apple stem pitting virus: Evidence of recombination. J. Plant Pathol..

[18] Boulila M. (2010). Putative recombination events and evolutionary history of five economically important viruses of fruit trees based on coat protein-encoding gene sequence analysis. Biochem. Genet..

[19] Jiang S., An H., Xu F., Zhang X. (2020). Chromosome-level genome assembly and annotation of the loquat (*Eriobotrya japonica*) genome. GigaScience.

[20] Stecher G., Tamura K., Kumar S. (2020). Molecular evolutionary genetics analysis (MEGA) for macOS. Mol. Biol. Evol..

[21] Nei M., Kumar S. (2000). Molecular Evolution and Phylogenetics.

[22] Tamura K. (1992). Estimation of the number of nucleotide substitutions when there are strong transition-transversion and G + C-content biases. Mol. Biol. Evol..

[23] Guindon S., Dufayard J.F., Lefort V., Anisimova M., Hordijk W., Gascuel O. (2010). New algorithms and methods to estimate maximum-likelihood phylogenies: Assessing the performance of PhyML 3.0. Syst. Biol..

[24] Jones D.T., Taylor W.R., Thornton J.M. (1992). The rapid generation of mutation data matrices from protein sequences. Comput. Appl. Biosci..

