# Supplementary material for: Loquat (Eriobotrya japonica) is a New Natural Host of Apple Stem Pitting Virus
Source: Plants (Basel). 2020 Nov 13;9(11):1560. doi: 10.3390/plants9111560 (PMC7696262; doi:10.3390/plants9111560)
Supplement: Supplementary file 1 [file plants-09-01560-s001.pdf]

Table S1. Accession numbers, host and origin for sequences used in the phylogenetic analysis performed on the CP N-terminal region

GenBank acc. number	Host	Country
ASJ27041	pear	Poland
ASJ27040	pear	Poland
ASJ27039	pear	Poland
ASJ27042	pear	Poland
ASJ27038	pear	Poland
ASJ27220	pear	Poland
ASJ27223	pear	Poland
ASJ27227	pear	Poland
ASJ27226	pear	Poland
ASJ27020	pear	Poland
ASJ27018	pear	Poland
ASJ27025	pear	Poland
ASJ27016	pear	Poland
ASJ27019	pear	Poland
ASJ27159	pear	Poland
ASJ26971	pear	Poland
ASJ26983	pear	Poland
AGR66264	pear	China
AGR66273	pear	China
ASJ26994	apple	Poland
AGR66254	pear	China
AVI26731	pear	China
ASJ26996	pear	Poland
SL61	loquat	Spain
ASJ26986	apple	Poland
ASJ27166	pear	Poland
ASJ27175	pear	Poland
ASJ27168	pear	Poland
ASJ26995	apple	Poland
ASJ27161	pear	Poland
ASJ27162	pear	Poland
AAK30566	pear	Poland
ASJ27167	pear	Poland
ASJ26987	apple	Poland
ASJ27218	pear	Poland
ASJ27221	pear	Poland
ASJ26981	pear	Poland
ASJ27001	pear	Poland
ASJ26992	apple	Poland
ASJ26997	pear	Poland
ASJ26982	pear	Poland
ASJ26999	pear	Poland
ASJ27002	pear	Poland
ASJ26998	pear	Poland
ASJ27036	pear	Poland
ASJ26978	pear	Poland
ASJ26985	pear	Poland
AGR66243	pear	China
AGR66262	pear	China
ASJ27200	pear	Poland
ASJ27202	pear	Poland
AGR66280	pear	China
AGR66242	pear	China
ASJ27190	pear	Poland
ASJ27188	pear	Poland
ASJ27187	pear	Poland
ASJ27196	pear	Poland
AGR66238	pear	China
AGR66261	pear	China
AGR66263	pear	China
AGR66249	pear	China
AGR66278	pear	China
AGR66277	pear	China
AGR66283	pear	China
AGR66266	pear	China
AAK30565	pear	Poland
ACD79976	pear	China
AGO50494	apple	South Korea
AID68942	apple	United Kingdom
ASJ27030	pear	Poland
ASJ27028	pear	Poland
ASJ27029	pear	Poland
ASJ27185	pear	Poland
ASJ27032	pear	Poland
ASJ27034	pear	Poland
ASJ27031	pear	Poland
CAI91566	apple	Czech Republic
ASJ27177	pear	Poland
ASJ27178	pear	Poland
ASJ27186	pear	Poland
AGR66258	pear	China
ASJ27179	pear	Poland
ASJ26991	apple	Poland
ASJ27182	pear	Poland
AGR66265	pear	China
AAK30563	pear	Poland
ASJ27198	pear	Poland
ASJ27208	pear	Poland
ASJ27199	pear	Poland
ASJ27216	pear	Poland
ASJ27209	pear	Poland
AGR66284	pear	China
AGR66245	pear	China
ASJ27184	pear	Poland
ASJ26988	apple	Poland
ASJ27035	pear	Poland
ASJ27219	pear	Poland
ASJ27026	pear	Poland
ASJ26989	apple	Poland
BBJ35813	pear	South Korea
AGU09697	apple	Germany
AGR66241	pear	China
ASJ27066	pear	Poland
ASJ27204	pear	Poland
ASJ27205	pear	Poland
ASJ26993	apple	Poland
ASJ27201	pear	Poland
ASS35979	apple	China
ASS35953	apple	China
ASS35963	apple	China
ASS35941	apple	China
ASS35975	apple	China
ASS35958	apple	China
ASS35965	apple	China
ASS35951	apple	China
ASS35945	apple	China
ALM89047	pear	India
AGR66239	pear	China
AGO50500	apple	South Korea
ASJ27152	pear	Poland
AGR66257	pear	China
ASJ26974	pear	Czech Republic
AGR66251	pear	China
ASJ27011	pear	Poland
ASJ27080	pear	Poland
ASS35962	apple	China
ASS35949	apple	China
ASJ27137	pear	Poland
ASJ27139	pear	Poland
QEY10274	pear	USA
AID68947	apple	Germany
QDK54728	apple	Brazil

GenBank acc. number	Host	Country
QKV49431	hawthorn	China
QDK54717	apple	Brazil
ATY47552	apple	China
AEP02959	pear	China
AEP02966	pear	China
AEP02964	pear	China
AWB36265	pear	China
AWB36266	pear	China
BBJ35808	pear	South Korea
AGR66246	pear	China
AGR66252	pear	China
ASJ27067	pear	Poland
ASJ27070	pear	Poland
ASJ27088	pear	Poland
ASJ27087	pear	Poland
ASJ27086	pear	Poland
ASJ27093	pear	Poland
AGR66281	pear	China
ASJ27072	pear	Poland
AGR66279	pear	China
AGO50493	apple	South Korea
AAM09801	apple	Poland
AAM14621	apple	Poland
ADI78887	apple	China
ASJ27140	pear	Poland
ASJ27116	pear	Poland
ASJ27097	pear	Poland
ASJ27068	pear	Poland
ASJ27096	pear	Poland
CBX25660	apple	India
QLQ34386	apple	India
ASJ27151	pear	Poland
ASJ27118	pear	Poland
ASJ27056	pear	Poland
ASJ27129	pear	Poland
ASS35954	apple	China
AHO49043	apple	China
ASJ27047	pear	Poland
ASJ27122	pear	Poland
ASJ27109	pear	Poland
ASJ27117	pear	Poland
ASJ27106	pear	Poland
ASJ27119	pear	Poland
ASJ27076	pear	Poland
AAK30564	pear	Poland
ASJ26984	pear	Poland
ASJ27107	pear	Poland
ASJ27114	pear	Poland
ASJ26973	apple	Poland
ASJ26979	pear	Poland
ASJ26975	pear	Poland
AIZ72683	apple	India
AGR66282	pear	China
ASJ27100	pear	Poland
ASJ27098	pear	Poland
ASS35977	apple	China
AGR66276	pear	China
AGR66274	pear	China
ASJ26980	pear	Poland
QEY10269	pear	USA
APT43452	pear	China
AGR66256	pear	China
ASJ27071	pear	Poland
ASJ27073	pear	Poland
ASJ27075	pear	Poland
AGR66248	pear	China
AGR66247	pear	China
AGR66259	apple	China

GenBank acc. number	Host	Country
ASJ27014	pear	Poland
ASJ27108	pear	Poland
AGR66268	pear	China
AGR66267	pear	China
AAM09799	pear	Poland
ASJ27059	pear	Poland
ASJ27115.1	pear	Poland
ASJ27012.1	pear	Poland
AGR66271.1	pear	China
ASJ27062.1	pear	Poland
AGR66240.1	pear	China
ASJ26990.1	apple	Poland
ASS35971.1	apple	China
ASJ27063.1	pear	Poland
ASJ27078.1	pear	Poland
ASJ27112.1	pear	Poland
AGR66255.1	pear	China
ASJ27124.1	pear	Poland
ASJ27007.1	pear	Poland
ASJ26976.1	pear	Poland
ASS35946.1	pear	China
ASJ27134.1	pear	Poland
ASJ27057.1	pear	Poland
AGR66244.1	pear	China
ASJ27006.1	pear	Poland
ASJ27008.1	pear	Poland
NP604468.1	pear	Unknown
ASJ27083.1	pear	Poland
ASJ27126.1	pear	Poland
ASJ27077.1	pear	Poland
ASS35976.1	apple	China
ASJ26977.1	pear	Poland
ADI78890.1	apple	China
BCB67679	apple	Japan
AUY61894	apple	China
AUY61889	apple	China
AGO50497.1	apple	South Korea
AAY21815	apple	Czech Republic
AGR66269	pear	China
ASS35974	apple	China
AGR66272	pear	China
AGR66275	pear	China
ASS35968	apple	China
ASJ27055	pear	Poland
ASJ27191	pear	Poland
ASJ27197	pear	Poland
AGR66260	apple	China
ASS35952	apple	China
ASS35957	apple	China
ADN92568	pear	Taiwan
ASS35955	apple	China
AAL32458	apple	Poland
ASJ27064	pear	Poland
AIW58880	apple	China
ASS35960	apple	China
ASS35942	apple	China
ASJ27054	pear	Poland
ASJ27052	pear	Poland
QDK54723	apple	Brazil
AGO50496	apple	South Korea
AGR66270	pear	China
ASJ27049	pear	Poland
QGH51263	apple	Germany
ASS35972	apple	China
ASJ27053	pear	Poland
ASS35964	apple	China
AGR66250	pear	China
ASJ27050	pear	Poland

GenBank acc. number	Host	Country
ACM69046	apple	China
ASS35969	apple	China
ASJ26972	pear	Czech Republic
ASS35978	apple	China
ACM69047	apple	China
ASJ27046	pear	Poland
QDK54733	apple	Brazil
AGR66237	pear	China
ASS35973	apple	China
ADI78889	apple	China
ASJ27165	pear	Polonia
AGO50495	apple	South Korea
ABY28337	apple	China
AAL32457	apple	Poland
AIZ72684	apple	India
AGO50499	apple	South Korea
ASJ27136	pear	Poland
ASJ27148	pear	Poland
AAM09800	apple	Poland
ASJ27138	pear	Poland
ASJ27141	pear	Poland
ASJ27146	pear	Poland
ADI78888	apple	China
AHF50171	apple	China
AGO50498	apple	South Korea
ASJ27149	pear	Poland
QNH86250	apple	India
ASJ27147	pear	Poland
ASS35943	apple	China
ASJ27145	pear	Poland
QNH86251	apple	India
CBA12158	apple	India
ASS35948	apple	China
ASS35967	apple	China
ASS35966	apple	China
